# Identification and quantification of cannabinol as a biomarker for local hemp retting in an ancient sedimentary record by HPTLC-ESI-MS

**DOI:** 10.1007/s00216-020-02492-0

**Published:** 2020-02-14

**Authors:** Theresa Schmidt, Annemarie Elisabeth Kramell, Florian Oehler, Ralph Kluge, Dieter Demske, Pavel E Tarasov, René Csuk

**Affiliations:** 1 Department of Organic Chemistry, Martin-Luther-University Halle-Wittenberg, Kurt-Mothes-Str. 2, 06120 Halle, Germany; 2 Department of Inorganic Chemistry, Martin-Luther-University Halle-Wittenberg, Kurt-Mothes-Str. 2, 06120 Halle, Germany; 3 Section Paleontology, Institute of Geological Sciences, Freie Universität Berlin, Malteserstr. 74-100, 12249 Berlin, Germany

**Keywords:** HPTLC, Cannabinol, Sediment, Biomarker, *Cannabis*, Hemp retting

## Abstract

*Cannabis* products have been used in various fields of everyday life for many centuries, and applications in folk medicine and textile production have been well-known for many centuries. For traditional textile production, hemp fibers were extracted from the stems by water retting in stagnant or slow-moving waters. During this procedure, parts of the plant material‚ among them phytocannabinoids‚ are released into the water. Cannabinol (CBN) is an important degradation product of the predominant phytocannabinoids found in *Cannabis* species. Thus, it is an excellent indicator for present as well as ancient hemp water retting. In this study, we developed and validated a simple and fast method for the determination of CBN in sediment samples using high-performance thin-layer chromatography (HPTLC) combined with electrospray ionization mass spectrometry (ESI-MS), thereby testing different extraction and cleanup procedures‚ as well as various sorbents and solvents for planar chromatography. This method shows a satisfactory overall analytical performance with an average recovery rate of 73%. Our protocol enabled qualitative and quantitative analyses of CBN in samples of a bottom sediment core‚ having been obtained from a small lake in Northern India, where intense local retting of hemp was suggested in the past. The analyses showed a maximum CBN content in pollen zone 4 covering a depth range of 262–209 cm, dating from approximately 480 BCE to 1050 CE. These findings correlate with existing records of *Cannabis*-type pollen. Thus, the method we propose is a helpful tool to track ancient hemp retting activities.

**Electronic supplementary material:**

The online version of this article (10.1007/s00216-020-02492-0) contains supplementary material, which is available to authorized users.

**Figure:**
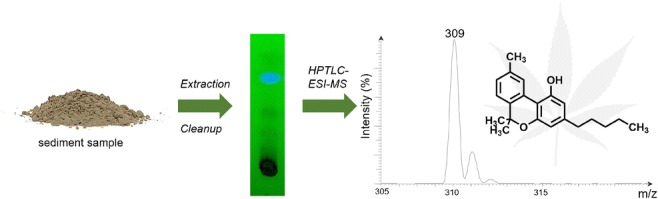
Graphical Abstract

## Introduction

**Fig. 1:**
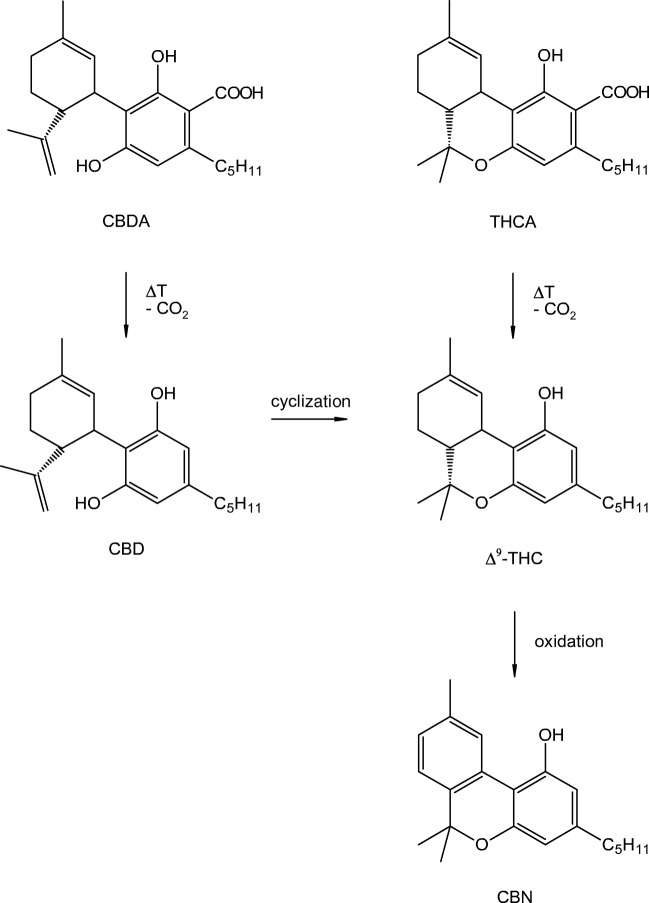
Conversion of CBDA and THCA to CBD and THC as well as the formation of the main degradation product CBN

*Cannabis* has been used by humans for many centuries and is probably one of the oldest cultivated plants [1]. It is widely distributed around the world, and archaeological finds indicate its usage for more than 2500 years. For instance, almost complete ancient *Cannabis* plants as well as parts of it have been excavated from different tombs in the Jiayi and Yanghai cemetery located in Northwestern China dating back to the first millennium BCE [2, 3]. *Cannabis* is a versatile plant and has been used as medicine, food source (seeds and oil), fuel, and psychedelic drug and also as a construction material or for the production of textiles and paper. The production of hemp fibers, e.g., for the manufacturing of robes, requires the separation of the fibers from the stems through microbiological and physical processes occurring during retting. Traditionally, the extraction of the fibers from stems is performed in stagnant or slow-moving waters, thereby submerging the stems in water for several days. During this treatment plant material and among other substances, phytocannabinoids are released into the waters. Phytocannabinoids are unique to the *Cannabis* species. The predominant phytocannabinoids in drug- and fiber-type *Cannabis* are (-)-∆^9^-*trans*-tetrahydrocannabinolic acid (THCA) and cannabidiolic acid (CBDA) that are transformed by a non-enzymatically decarboxylation upon heating after harvesting and during storage into their corresponding neutral forms, namely (-)-∆^9^-*trans*-tetrahydrocannabinol (∆^9^-THC) and cannabidiol (CBD). Phytocannabinoids accumulate in female flowers and in most aerial parts of the plant. In contrast, *Cannabis* pollen, seeds, and roots contain only low concentrations of these compounds [4]. The concentration of cannabinoids and the ratio ∆^9^-THC:CBD depend on different parameters such as growth conditions, variety, age, harvest time, and storage conditions [4, 5]. Especially during storage, ∆^9^-THC is relatively unstable whenever *Cannabis* products such as flowering tops, oils, and resins are exposed to air, light, heat, or acidic conditions [6]. Eventually, cannabinol (CBN) is one of the most important products of degradation [7, 8]. CBD also undergoes changes during long-term storage, e.g., the transformation to ∆^9^-THC by an acid-catalyzed cyclization, followed by the decay of ∆^9^-THC to CBN (Fig. 1) [8, 9]. Therefore, it is not surprising that CBN has been detected as the major degradation product of cannabinoids in dried *Cannabis* flowers dating from around 1896–1905 [10]. Studies concerning the CBN content in sedimentary records, however, are rare. Thus, Lavrieux et al. reported about the detection of CBN, preserved in sediment samples from lake Aydat in the French Massif Central, covering the past 1800 years [11], thereby relating the presence of CBN to the retting of locally grown *Cannabis* plants for fiber production. This finding was supported by the analysis of pollen and historical data. Thus, determination of CBN contents in sedimentary cores seems to be an excellent possibility to trace ancient water retting activities.

In a previous study, sediment samples from lake Aydat were investigated, and the detection of CBN was performed by gas chromatography mass spectrometry (GC-MS). However, this approach required a time-consuming derivatization with *N*,*O*-bis(trimethylsilyl)trifluoroacetamide (BSTFA). In general, different methods, e.g., GC-MS, liquid chromatography tandem mass spectrometry (LC-MS/MS), or (high-performance) thin-layer chromatography MS ((HP)TLC-MS) technique, are available for the quantification of cannabinoids in various matrices such as human blood, plasma, hair, urine, rodent tissues, or plant material [12–14]. Here, HPTLC combined with MS detection is a versatile and useful tool for the analysis of complex compounds in challenging matrices, thus enabling a relatively simple, rapid, and inexpensive qualitative and quantitative determination. However, the application of HPTLC-MS for the determination of CBN in sediment samples, a very complex matrix, has not yet been described so far.

In this study, we report on the development and validation of a HPTLC-electrospray ionization (ESI)-MS method for the identification and quantification of CBN as a molecular biomarker for ancient hemp retting in sedimentary records allowing a fast and accurate high-throughput screening of sediment samples. In this context, samples of a sediment core from a small lake in Northern India, covering a period from 2220 BCE to 1390 CE, were tested. Previous studies on these samples have already shown high percentages of *Cannabis*-type pollen, thus indicating an intense local retting of hemp [15].

## Material and methods

### Chemicals and materials

CBN (1 mg/mL in methanol, certified reference material), CBN-d_3_ (100 μg/mL in methanol, certified reference material), CBD (1 mg/mL in methanol, certified reference material), and CBD-d_3_ (100 μg/mL in methanol, certified reference material) were bought from Cerilliant; acetonitrile (HPLC gradient grade), methanol (HPLC gradient grade), *n*-hexane (HPLC grade), *n*-heptane (HPLC grade), and formic acid (98–100%) were obtained from VWR Chemicals; dichloromethane (HPLC grade) from Carl Roth, triethylamine from ACROS Organics, Fast Blue Salt B (FBS, dye content ~ 95%) from Sigma-Aldrich, Chromabond SiOH (1 ml/100 mg), Chromabond C_18_ ec (1 ml/ 100 mg) as well as TLC (thin-layer chromatography) plates (silica gel 60, ALUGRAM Xtra SIL G UV_254_ and octadecyl-modified silica, ALUGRAM RP-18 W/UV_254_) from Macherey-Nagel, TLC plates (silica gel 60 without fluorescent indicator on aluminum sheets), and HPTLC (high-performance thin-layer chromatography) plates (silica gel 60 F_254_ MS-grade for matrix-assisted laser desorption/ionization (MALDI) and silica gel 60 F_254_ on glass plates) were purchased from Merck KGaA and analytical sea sand from Grüssig GmbH. Distilled diethyl ether and acetone were produced with a rotary evaporator from BÜCHI.

### Preparation of standard solutions

A stock solution of CBN from Cerilliant (1 mg/mL in methanol) was diluted with methanol to obtain working solutions down to a concentration of 0.3 μg/mL. CBN-d_3_ working solutions (2.5 μg/mL and 5.0 μg/mL) as internal standards were prepared in methanol. For the calibration, solutions holding different mixtures of CBN and CBN-d_3_ were prepared (CBN, in the range of 1.0–6.2 μg/mL; CBN-d_3_, 2.5 μg/mL), and for the validation of the HPTLC-ESI-MS method, different mixtures of CBN and CBN-d_3_ were used (CBN, 1.4, 1.8, 2.2, 2.5, 3.4, 5.0, 5.4 μg/mL; CBN-d_3_, 2.5 μg/mL).

### Detection of CBN with FBS reagent (modified according to an application note from CAMAG [16])

The post-chromatographic detection reaction was performed with FBS reagent using CBN working solutions and sample extracts (25 μL aliquots) spotted onto TLC or HPTLC plates. For the preparation of the FBS reagent, FBS (250 mg) was completely dissolved in distilled water (10 mL) and was mixed with methanol (25 mL) and dichloromethane (15 mL). This reagent was always freshly prepared before use. (HP)TLC plates were developed in *n*-hexane/acetone/triethylamine (40:20:2 v/v/v; see Watanabe et al. [17]), sprayed with the reagent, and the presence of red spots indicated a positive response.

### Detection of CBN with cerium-molybdenum reagent

In addition, post-chromatographic detection reactions were performed with cerium-molybdenum reagent using CBN working solutions and sample extracts (25 μL aliquots) spotted onto (HP)TLC plates. For the preparation of the cerium-molybdenum reagent, cerium(IV) sulfate (400 mg) and ammonium molybdate (20 g) were dissolved in 10% (v/v) sulfuric acid (400 mL). (HP)TLC plates were developed in *n*-heptane/diethyl ether/formic acid (75:25:0.3 v/v/v; according to an application note from CAMAG [16]), *n*-heptane/diethyl ether (90:10 v/v), or *n*-hexane/acetone/triethylamine (40:20:2 v/v/v), sprayed with the reagent, and blue spots became visible after exposure to heat.

### Samples

Samples of a 3.55-m-long sediment core from Badanital (30° 29′ 50″ N, 78° 55′ 26 E, 2083 m a.s.l.), a small lake in the West Himalayan oak forest zone in Northern India, have been investigated. The core was retrieved using a piston corer in January 2008 (see Kotlia and Joshi [18]). Contiguous subsamples were taken in 1 cm slices and dried at 30 °C for storage and transportation to the pollen laboratory at the Institute of Geological Sciences, Freie Universität Berlin. Sediment samples, covering a period from 2220 BCE to 1390 CE, were examined concerning *Cannabis* pollen; they were categorized into real samples (positive samples), containing *Cannabis* pollen, and negative samples without *Cannabis* pollen. The procedure for pollen analyses is based on morphological characteristics as well as further results of the determination of palynomorphs; detailed results from accelerator mass spectrometry (AMS) radiocarbon dating using bulk sediment rich in organics were described by Demske et al. [15]. For the reconstruction of climatic changes based on geochemical parameters of sediment samples from Badanital lake, see Kotlia and Joshi [18].

### HPTLC-ESI-MS analysis

Standard solutions and the extracts were spotted onto the TLC or HPTLC plates as 2 mm bands, in 25 μL aliquots, 20 mm from the bottom edge and 8 mm apart using a Linomat 5 (CAMAG, Switzerland). Plates were developed in a rectangular TLC developing chamber to a distance of 50 mm in 15 min using *n*-heptane/diethyl ether (90:10 v/v) as the developing solvent. For the optimization of the chromatographic separation, TLC and HPTLC plates as well as various developing solvents were tested, e.g., *n*-heptane/diethyl ether/formic acid (75:25:0.3 v/v/v) or *n*-hexane/acetone/triethylamine (40:20:2 v/v/v).

HPTLC plates were inspected both under white light and with under UV light at λ = 254 nm. Beside investigations by MS, different spray reagents were also used for the detection of CBN (see detection of CBN with FBS and cerium-molybdenum reagent).

A TLC-MS interface (Plate Express from Advion combined with an isocratic pump) was utilized for the elution of compounds from the HPTLC plates into an expression^L^ CMS (compact mass spectrometer from Advion, UK) system, equipped with an ESI ion source (negative mode, capillary temperature 250 °C, capillary voltage 180 V, source voltage offset 20, source voltage span 30, ESI source voltage 2500 V, source gas temperature 200 °C, MS scan range 200–400 *m/z*). Prior to the measurements, substance-specific parameters were determined by direct inlet of CBN working solutions. Methanol was used as eluent (flow rate 0.2 mL/min).

### Offline HPTLC-ESI-HRMS analysis

Standard solutions were spotted onto HPTLC plates; HPTLC plates were developed in *n*-heptane/diethyl ether (90:10 v/v), and after 5 h, spots were marked; stationary phase was scraped from the plates and compounds were eluted with methanol (1 mL). This solution was filtered (0.2 μm PTFE) and utilized for HRMS experiments using a Q Exactive Plus mass spectrometer from Thermo Scientific equipped with an ESI ion source (negative mode, spray voltage 3287 V, spray current 1 μA, capillary temperature 320 °C, sheath gas flow rate 10 L/min, MS scan range 200–900 *m/z*).

### Sample extraction and preparation

**Fig. 2:**
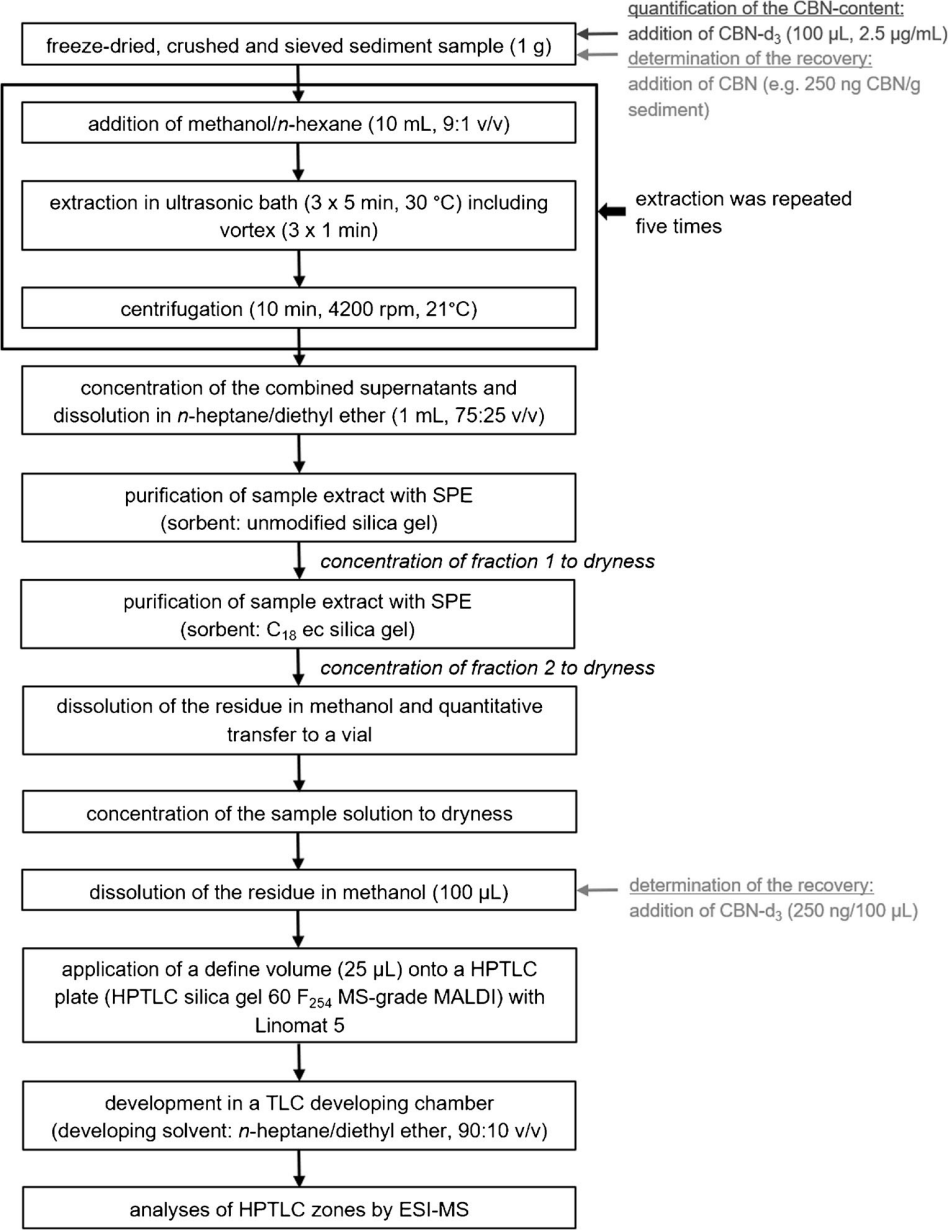
Flow diagram for the extraction of the samples and cleanup protocol. For the quantification of the CBN content, internal standard CBN-d_3_ was added after sample has been weighed. For the determination of the recovery, CBN-spiked sediment samples (approximately 140, 250, and 330 ng CBN/g sediment) were used, and the residue of the purification with reversed-phase sorbent was solved in a methanolic solution of CBN-d_3_ (100 μL, 2.5 μg/mL) after having been transferred to a vial and before spotting the solution onto a HPTLC plate

The remaining sediment samples analyzed for pollen were sent to the Department of Organic Chemistry, Martin-Luther-University Halle-Wittenberg (Halle), and used in the current study. An aliquot (1 g) of each sample was extracted with methanol/hexane (10 mL, 9:1 v/v) by the following procedure: 1 min on a vortex and 15 min ultrasonic bath at 30 °C including vortex again after 5 and 10 min. Subsequently, the suspension was centrifuged (10 min, 21 °C, 4200 rpm) in a centrifuge 5403 from Eppendorf. The extraction of the sample was repeated five times. The supernatants were combined, and the solution was evaporated to dryness on a rotary evaporator (temperature of the water bath, 30 °C). The residue was dissolved in *n*-heptane/diethyl ether (1 mL, 75:25 v/v) with the help of an ultrasonic bath at 30 °C for a few seconds. Afterwards, the sample extract was transferred to a Chromabond SiOH column conditioned with *n*-heptane/diethyl ether (75:25 v/v), the sample container was rinsed with *n*-heptane/diethyl ether (3 × 1 mL, 75:25 v/v), the rinse solution was also transferred onto the sorbent, and the analyte was eluted with *n*-heptane/diethyl ether (2 mL, 75:25 v/v). The eluate (fraction 1: combined solutions, approximately 6 mL) was evaporated to dryness on a rotary evaporator (temperature of the water bath, 30 °C), and the residue was dissolved in acetonitrile/water (500 μL, 70:30 v/v). The solution was transferred to a Chromabond C_18_ ec column conditioned with acetonitrile/water (70:30 v/v). The sample vessel was rinsed with acetonitrile/water (1 mL, 70:30 v/v), the rinse solution was also transferred onto the sorbent, and the analyte was eluted with acetonitrile/water (500 μL, 70:30 v/v, and 3 mL, 80:20 v/v). The eluate (fraction 2: combined solutions, approximately 5 mL) was evaporated to dryness on a rotary evaporator (temperature of the water bath, 50 °C). The residue was dissolved in methanol (1 mL) and transferred to a vial. The sample pot was rinsed with methanol (3 mL), and this solution was also transferred to this vial step-by-step. The sample solution in the vial was concentrated to dryness in a stream of argon with heat from a laboratory sand bath at 70 °C. The residue was solved in methanol (100 μL), and a defined volume (25 μL) of the sample extract was spotted onto a HPTLC plate (Fig. 2).

For the optimization of the extraction procedure, various extraction times and further extracting agents were tested, e.g., dichloromethane/methanol (90:10 v/v and 1:1 v/v).

### Determination of sediment pH

For the determination of the pH of a sample, a pH electrode from HANNA instruments was used. An aliquot (0.5 g) of the sediment sample was suspended in a solution of calcium chloride (0.01 m) at the ratio of 1:2.5. After the sedimentation, the pH was measured.

### Determination of the loss on ignition (modified according to Heiri et al. [19])

The loss on ignition at 550 °C (LOI_550_) was determined using a thermobalance STA 449C from Netzsch (reference: empty crucible of corundum). An aliquot of the sample (15–25 mg) was weighted in a crucible of corundum, and a stream of gas (N_2_/O_2_ = 80/20, 50 mL/min) was applied. Before each measurement, an equilibration of the thermobalance was performed for about 30 min at room temperature with current gas flow. Afterwards, the sample chamber was heated to 800 °C (heat rate 10 K/min). The weight loss is proportional to the amount of organic carbon contained in samples. For the calculation, the following formula (1) was used:

1 $$ {LOI}_{550}=\left(\left({DW}_{105}-{DW}_{550}\right)/{DW}_{105}\right)\times 100 $$

where LOI_550_ represents the LOI at 550 °C (as percentage), DW_105_ correspond to the dry weight of the sample before combustion, and DW_550_ to the dry weight of sample after heating to 550 °C (both in mg, see Heiri et al. [19]).

### Determination of C/N content

The determination of the C and N content of the samples were performed with the analytical instrument “Vario EL” from the company Elementar.

### Validation of the HPTLC-ESI-MS method

#### Specificity

The test for specificity was performed with a negative sample (without CBN) and a real sample (containing CBN) applying the post-chromatographic detection reaction with cerium-molybdenum reagent and HPTLC-ESI-MS using standard compounds. For HPTLC-ESI-MS experiments as well as for the detection of CBN with the cerium-molybdenum reagent, a sample extract was spotted onto a HPTLC plate; the HPTLC plate was developed in *n*-heptane/diethyl ether (90:10 v/v) and investigated by HPTLC-ESI-MS or sprayed with cerium-molybdenum reagent.

#### Linearity, limit of detection, and limit of quantification

The linearity of the calibration function was tested with the Mandel’s test. Limit of detection (LOD) and limit of quantification (LOQ) were determined by means of a calibration curve method according to DIN 32645 [20], thereby spotting the calibration solutions (see preparation of standard solutions) onto HPTLC plates; the HPTLC plates were developed using *n*-heptane/diethyl ether (90:10 v/v) and investigated by HPTLC-ESI-MS. Each calibration solution was measured three times, and analyses were executed with average peak areas of the mass peaks of CBN (sum of *m/z* 309 and 354) and CBN-d_3_ (sum of *m/z* 312 and 357). Average peak area ratios and CBN concentrations are depicted in Fig. S1 and Tab. S1 (see Electronic Supplementary Material, ESM).

#### Precision

For determining the repeatability, two to five replicate determinations on eight different days were carried out. For this purpose, a solution containing CBN and CBN-d_3_ (CBN 3.4 μg/mL, CBN-d_3_ 2.5 μg/mL) was spotted onto HPTLC plates; HPTLC plates were developed using *n*-heptane/diethyl ether (90:10 v/v) and investigated by HPTLC-ESI-MS. For the interpretation of the repeatability, the relative standard deviation (RSD) of the CBN content was used. Furthermore, Dixon’s Q test and Neumann trend test were applied for the identification of outliers or trends (see Table S2 of the ESM).

For determining the method precision, sediment sample BT-78 was utilized. Analyses were carried out with a number of six replicates and each aliquot of the sediment sample (approximately 1 g) was spiked with a defined concentration of CBN (100 μL; 3.4 μg/mL) and CBN-d_3_ (100 μL; 2.5 μg/mL). Extractions and preparations of the different samples were performed independently of each other as described above. After SPE using a Chromabond C_18_ ec column, the residues were dissolved in methanol (100 μL) and defined volumes (25 μL) of the sample extracts were spotted onto HPTLC plates; HPTLC plates were developed in *n*-heptane/diethyl ether (90:10 v/v) and were investigated by HPTLC-ESI-MS. For the interpretation of the method precision, the relative standard deviation (RSD) of the CBN content was used. Furthermore, Dixon’s Q test and Neumann trend test were applied for the identification of outliers or trends (see Table S2 of the ESM).

#### Trueness

The trueness was expressed in terms of recovery and bias. Bias calculation was performed for two concentration levels with a number of three replicates at each concentration (see Table S3 of the ESM). Sediment samples (approximately 1 g) were spiked with a defined concentration of CBN (100 μL; 5.4 μg/mL and 1.8 μg/mL) and CBN-d_3_ (100 μL; 2.5 μg/mL). Extractions and preparations of the different samples as well as the planar chromatographic separations were performed independently of each other as described above. Real samples were used for bias calculation due to a limited sample amount. For the bias calculation, the following formula (2) was used:

2 $$ \hat{\delta}=\overline{x}-T $$

where $\hat{\delta}$ represents the bias and *T* correspond to the “true” concentration and $\overline{x}$ to the mean value of the determined concentrations of the spiked sample materials.

#### Recovery

Three negative samples from different positions in the sedimentary core were used for the determination of the recovery. After the sediment samples have been weighed (approximately 1 g, see Fig. 2), defined concentrations of CBN (100 μL; 1.4, 2.5, and 3.4 μg/mL) were added and each concentration level was analyzed in duplicate (see Table S4 of the ESM). Extractions and preparations of the different samples were performed independently of each other on different days as described above. After SPE using a Chromabond C_18_ ec column, the residues were dissolved in a methanolic solution of CBN-d_3_ (100 μL; 2.5 μg/mL) and were spotted onto HPTLC plates; HPTLC plates were developed in *n*-heptane/diethyl ether (90:10 v/v) and were investigated by HPTLC-ESI-MS. The calculated CBN contents were compared with the target concentrations and the recovery rate was determined.

#### Stability of the standards

For testing the storage stability of standard solutions, solutions of CBN (50 and 100 μg/mL) were stored at − 12 °C in the dark, at room temperature (average temperature + 28 °C) in the dark and at room temperature exposed to sunlight. The different solutions were examined over a 4-week period. For analyses, solutions of CBN were diluted, CBN-d_3_ was added, and the mixtures (CBN 2.2 and 5.0 μg/mL; CBN-d_3_ 2.5 μg/mL) were spotted onto HPTLC plates. Mass peak areas of CBN (sum of *m/z* 309 and 354) and CBN-d_3_ (sum of *m/z* 312 and 357) were utilized for calculating the stability of CBN. For the identification of trends, a trend test by Neumann [20] was performed (see Table S5 of the ESM).

In addition, the stability of CBN and CBN-d_3_ already having been spotted onto (HP)TLC plates was evaluated. For this purpose, a solution containing CBN and CBN-d_3_ (CBN 5.0 μg/mL; CBN-d_3_ 5.0 μg/mL) was spotted in triplicate onto a TLC and a HPTLC plate. (HP)TLC plates were developed in *n*-heptane/diethyl ether (90:10 v/v) and were investigated by HPTLC-ESI-MS at a time interval of 3 h (measuring after 0.5, 1.5, and 3 h). Furthermore, investigations were performed on (HP)TLC plates spotted with CBN and CBN-d_3_ without developing the chromatograms. For analyses, mass peak intensities of CBN (ratios of *m/z* 309 and 354) and CBN-d_3_ (ratios of *m/z* 312 and 357) were utilized.

## Results and discussion

### Development of a HPTLC-ESI-MS method for the identification and quantification of CBN in sediment samples

**Fig. 3:**
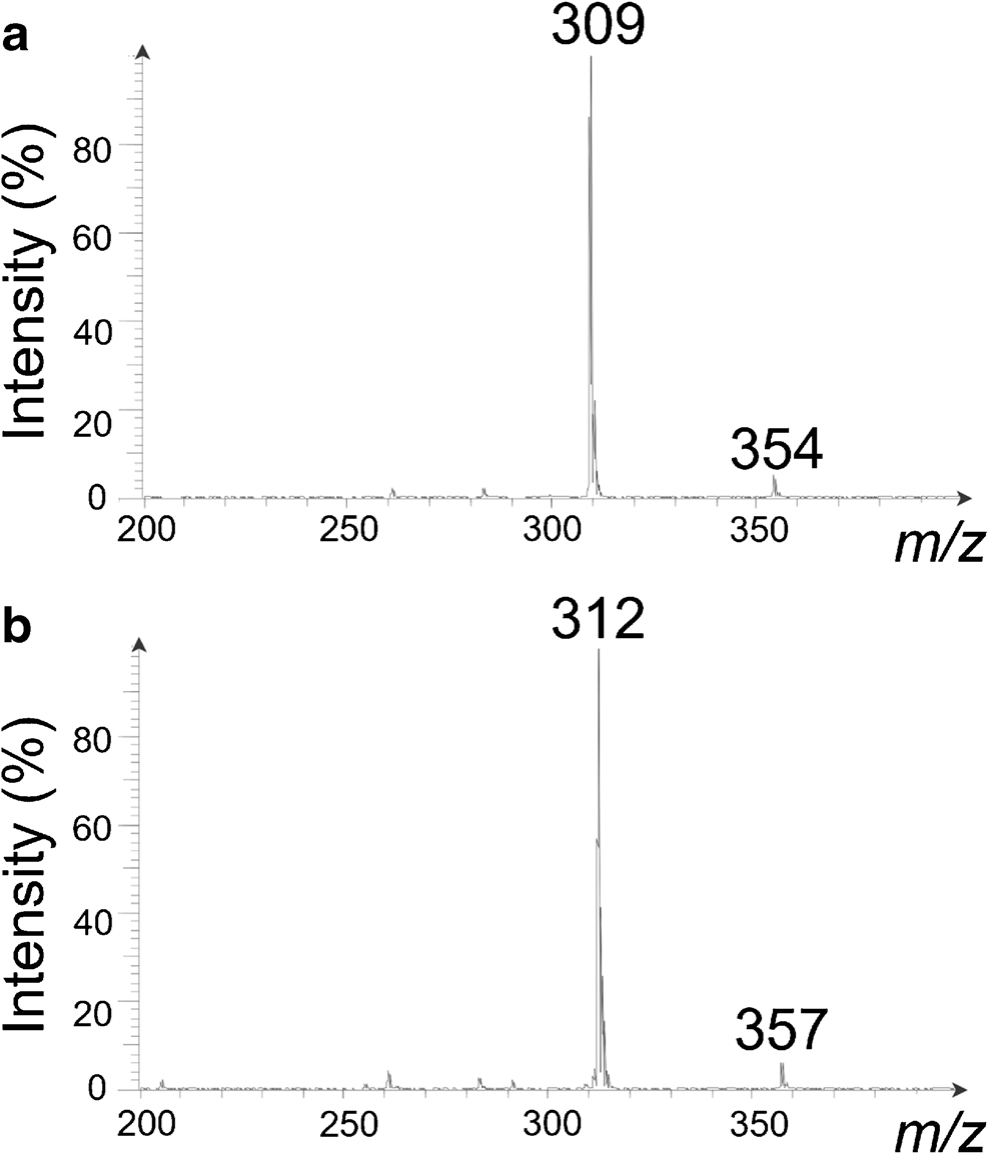
MS spectrum of **a** CBN and **b** CBN-d_3_ spotted onto a HPTLC silica gel 60 plate recorded immediately after chromatographic separation using *n*-heptane/diethyl ether (90:10 v/v) as developing solvent

Determination of CBN content in sediment samples was performed by HPTLC-ESI-MS. For this purpose, sediment samples were extracted followed by a subsequent elimination of disturbing matrix compounds using an orthogonal SPE sample preparation. Afterwards, purified extracts were spotted onto HPTLC plates, the HPTLC plates were developed and the HPTLC zones were analyzed by ESI-MS (MS scan range *m/z* 200–400). Mass spectra of standards eluted from HPTLC silica gel 60 plates show intense peaks at *m/z* 309 and 312, assigned to the quasi-molecular ions [M-H]^−^ of CBN and CBN-d_3_. In addition, extra mass peaks at *m/z* 354 (working solutions of CBN spotted onto HPTLC plates) and 357 (working solutions of CBN-d_3_ spotted onto HPTLC plates) were observed (Fig. 3). These signals appear after the application of CBN and CBN-d_3_ onto TLC or HPTLC silica gel 60 plates and were not found in fresh or aged (for 24 h) methanolic working solutions of CBN and CBN-d_3_ or in the context of blanks (blanks for the whole method as well as investigations on TLC or HPTLC plates using different developing solvents without the addition of CBN or CBN-d_3_). However, after development of the (HP)TLC plates, the proportion of these extra peaks increased compared with investigations without contact to a developing solvent. In this context, different developing solvents and stationary phases (octadecyl-modified TLC silica layers, unmodified (HP)TLC silica layers with and without fluorescent indicator on aluminum sheets and glass plates) were tested. Mass spectra of standards eluted from octadecyl-modified TLC silica layers show peaks at *m/z* 309 and 312. However, no signals at *m/z* 354 or 357 were observed. In contrast, in all experiments performed on unmodified silica layers, extra mass peaks at *m/z* 354 and 357 were detected. Furthermore, the proportion of these peaks increased in time. Immediately after application of the cannabinoids and the development of the HPTLC silica gel 60 plates, peak intensity ratios of *m/z* 309/354 and 312/357 were 10.1 (8.01E7/7.91E6) and 8.3 (3.65E7/4.41E6, see Fig. S2a of the ESM). After 3 h, the ratios were in either case 0.3 (1.67E7/5.08E7 and 7.62E6/2.61E7, see Fig. S2b of the ESM). Thus, investigations were performed immediately after application and chromatographic separation and signals at *m/z* 354 and 357 were included in the quantitative determination of CBN.

Furthermore, methanolic working solutions of CBD and CBD-d_3_ were spotted onto TLC or HPTLC silica gel 60 plates. After the development of the plates, besides intense peaks at *m/z* 313 and 316 (assigned to the quasi-molecular ions [M-H]^−^), extra peaks with a mass shift of 45 were also detected at *m/z* 358 and 361 (see Fig. S3a and S3b of the ESM). As indicated by TLC and HRMS studies on CBN and CBN-d_3_, the mass shift of 45 points to the presence of transient adducts with the silica gel from the plates. This will be subject to further studies.

In addition, different spray reagents were tested to find a pretest confirming the presence of CBN in sediment samples by a color reaction.

#### Optimization of mobile and stationary phase for planar chromatography

For the optimization of the planar chromatographic separation, different sorbents and solvents were tested. An acceptable separation of CBN and matrix compounds was found on TLC silica gel 60 sorbent using *n*-heptane/diethyl ether/formic acid (75:25:0.3 v/v/v) or *n*-hexane/acetone/triethylamine (40:20:2 v/v/v) as developing solvent (see Fig. S4 of the ESM). However, bands were rather diffused. Therefore, normal phase HPTLC on silica gel 60 was used to achieve sharper bands, thereby obtaining a satisfactory resolution of CBN and matrix compounds using *n*-heptane/diethyl ether (90:10 v/v) as the mobile phase.

#### Optimization of sample extraction and preparation

Different extracting agents and extraction times were tested using analytical sea sand spiked with CBN due to a limited sample amount (Table 1). An extraction in an ultrasonic bath with methanol/*n*-hexane (90:10 v/v) for 5 × 15 min provided satisfactory results concerning the recovery of CBN (recovery rate 97%). Sample handling was also optimized with CBN spiked sea sand samples.

**Table 1:** Optimization of sample extraction using different extracting agents and analytical sea sand spiked with CBN (extraction time 5 × 15 min)

Extracting agent	Recovery (%)
Methanol/*n*-hexane (90:10 v/v)	97
Dichloromethane/methanol (90:10 v/v)	90

For the determination of CBN in sediment matrices (extracts of real and spiked negative samples), two SPE columns were combined. Matrix simplification was carried out with a combination of normal phase and reversed phase sorbents. Some losses of CBN during this two-step purification procedure were eliminated through fine-tuning (recovery rate of CBN for the whole procedure including extraction and purification: see validation results).

#### Detection of CBN by spray reagents

**Fig. 4:**
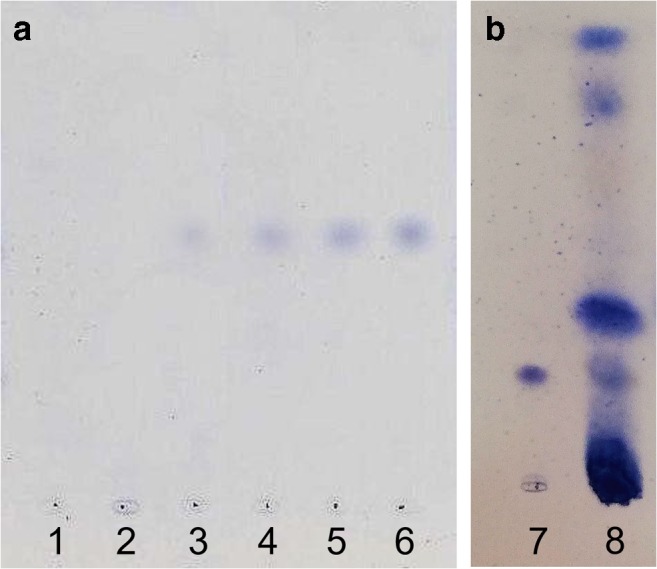
HPTLC silica gel 60 plates developed using **a***n*-heptane/diethyl ether/formic acid (75:25:0.3 v/v/v) or **b***n*-heptane/diethyl ether (90:10 v/v) as developing solvent after derivatization with cerium-molybdenum reagent; observed under white light. Tracks 1–7 = CBN standard (from left to right increasing CBN concentration: 7.5, 15, 25, 55, 85, 125, 190 ng CBN/HPTLC zone); 8 = extract (extracting agent: methanol/hexane (10 mL, 9:1 v/v)) of real sample BT-96 spiked with CBN-d_3_ (62.5 ng CBN-d_3_/HPTLC zone)

**Fig. 5:**
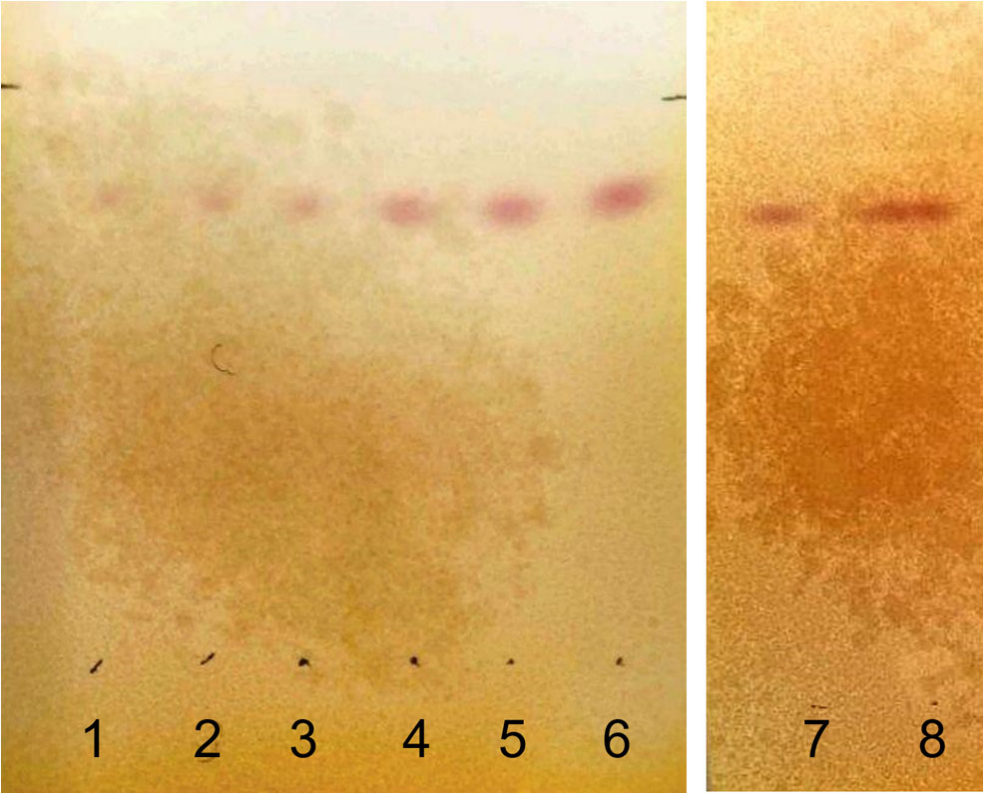
HPTLC silica gel 60 plates developed using *n*-hexane/acetone/triethylamine (40:20:2 v/v/v) as developing solvent after derivatization with FBS reagent; observed under white light. Tracks 1–7 = CBN standard (from left to right increasing CBN concentration: 7.5, 15, 25, 55, 85, 125, 190 ng CBN/HPTLC zone); 8 = extract (extracting agent: methanol/hexane (10 mL, 9:1 v/v)) of real sample BT-96 spiked with CBN-d_3_ (62.5 ng CBN-d_3_/HPTLC zone)

Different spray reagents were tested to confirm the presence of CBN in the sediment samples. The cerium-molybdenum reagent is suitable for the detection of low CBN concentrations up to 25.0 ng CBN/HPTLC zone using *n*-heptane/diethyl ether/formic acid (75:25:0.3 v/v/v, Fig. 4), *n*-heptane/diethyl ether (90:10 v/v), or *n*-hexane/acetone/triethylamine (40:20:2 v/v/v). CBN concentrations up to 7.5 ng CBN/HPTLC zone were detectable with the FBS reagent using *n*-hexane/acetone/triethylamine (40:20:2 v/v/v, Fig. 5). In the presence of the examined sediment matrices, exclusively CBN reacts under the described conditions with FBS resulting in red bands (FBS is known as a selective detection reagent for cannabinoids, see Fischedick et al. [21–23]). Thus, a post-chromatographic detection with FBS reagent was found to be appropriate as a pretest to confirm the presence of low CBN concentrations in sediment samples.

R_F_ values of CBN obtained by developing TLC or HPTLC plates in *n*-heptane/diethyl ether/formic acid (75:25:0.3 v/v/v), *n*-heptane/diethyl ether (90:10 v/v), and *n*-hexane/acetone/triethylamine (40:20:2 v/v/v) as well as observed colors of the bands are summarized in Table 2.

**Table 2:** Chromatographic data for CBN and observed colors of the bands using different spray reagents

Developing solvent	R_F_ value		Band color	
	TLC plate	HPTLC plate	Cerium-molybdenum reagent	FBS reagent
*n*-Heptane/diethyl ether/formic acid (75:25:0.3 v/v/v)	0.4	0.5	Blue	None
*n*-Heptane/diethyl ether (90:10 v/v)	–	0.2		
*n*-Hexane/acetone/triethylamine (40:20:2 v/v/v)	0.6	0.8		Red

### Validation results of HPTLC-ESI-MS method

Linearity, LOD, and LOQ were evaluated for CBN after chromatographic separation. The results from validation are summarized in Table 3 (further data see Table S1 and Fig. S1 of the ESM). LOD and LOQ were considered adequate for the purposes of the present study.

**Table 3:** Linearity, LOD, and LOQ for CBN determination by HPTLC-ESI-MS (tv, test value; cv, characteristic value)

Key figures	Results
Range (ng CBN/HPTLC zone)	25–155
*r*^2^ (coefficient of determination)	0.9979
Sy (residual standard deviation)	0.034
Sx (standard deviation for the method)	1.991
Residuals are normally distributed (R/s test, 99%)	Yes (tv = 3.32; cv = 2.86–4.34)
Residuals show a trend (Neumann trend test, 99%)	No (tv = 2.73; cv = 0.89)
Equation curve	y(x) = 0.0172x + 0.0245 y: peak area ratio of CBN and CBN-d_3_ x: concentration of CBN (ng CBN/ HPTLC zone)
LOD (ng CBN/ HPTLC zone)	6.4
LOQ (ng CBN/ HPTLC zone)	20.7
Results of the Mandel’s test according to DIN 32645	
Optimal regression model	Linear (tv = 0.02; cv = 9.64)
Linear regression acceptable	Yes

The developed HPTLC-ESI-MS method is specific for the determination of CBN in sediment samples. Post-chromatographic detection reaction with cerium-molybdenum reagent as well as the HPTLC-ESI-MS analyses on negative samples and real samples showed that CBN can be qualified and quantified even in the presence of interfering matrix components. Other related compounds being present in the complex sediment samples did not interfere with CBN during HPTLC-ESI-MS analyses. The method precision and the repeatability were determined with a RSD of 4.1% and 4.3% (see Table S2 of the ESM), thereby classifying only one measured value as outliner by Dixon’s test and was removed from the evaluation. The recovery rate of CBN after sample extraction and the described cleanup procedure (see sample extraction and preparation), determined with spiked negative samples from different positions in the sedimentary core, ranged from 63 to 82%, with an average recovery rate of 73% (see Table S4 of the ESM). Furthermore, no statistical difference has been observed between the mean and the “true” values (see bias calculation and Table S3 of the ESM). The storage stability of standard CBN solutions (2.2 and 5.0 μg/mL) was evaluated under different conditions (at − 12 °C in the dark, at room temperature in the dark, and exposed to sunlight). The tests showed that the concentration of CBN did not decrease more than 72% after 4 weeks (see Fig. S5 of the ESM). However, CBN underwent minor losses, maximal 84%, at a higher concentration of 5.0 μg/mL. Furthermore, no trend is discernible using a Neumann trend test.

In addition, it is conceivable to dissolve residues before the application onto HPTLC plates in a smaller volume of solvent (e.g., 30 μL instead of 100 μL) for increasing the concentration of the extract. This could enable a characterization of CBN in very low concentrations.

Thus, the method gives satisfactory results and allowed an appropriate examination of the CBN content in sediment samples.

### Analysis of sediment samples

The quantitative determination of CBN in sediment samples was performed by HPTLC-ESI-MS. In this context, a series of twelve sediment samples of one core from a small lake in the Garhwal Himalaya, referred to as Badanital lake, was analyzed in duplicate. The results as well as the sample position in the sedimentary core and estimated ages (for age estimation based on radiocarbon dating, see Demske et al. [15]) of the investigated samples are summarized in Table 4. Prior to our investigations, palynological analyses were performed (for characteristics of the respective pollen zones (PZ), see Demske et al. [15]), and *Cannabis* pollen were proven in the sample material; these samples were categorized as real (positive) samples. In contrast, samples holding no *Cannabis* pollen were categorized as negative samples. For these negative samples, the absence of CBN was assumed. Post-chromatographic detection reactions with FBS as well as HPTLC-ESI-MS studies confirmed the absence of CBN or a CBN content lower than 6.4 ng CBN/HPTLC zone (LOD), i.e., 25.6 ng CBN/g sediment.

**Table 4:** CBN content of sediment samples and characteristics of these samples. Analyses of the CBN content including the described extraction and cleanup procedure were performed in duplicate

Estimated ages (ca cal years BP)	Sample ID	Depth in sedimentary core (cm)	pHCaCl_2_	C content (%)	N content (%)	C/N (atomic)	LOI_550_ (%)	Average CBN content (ng CBN/HPTLC zone)	Average CBN content (ng CBN/g sediment)	PZ
Negative samples, without *Cannabis* pollen										
580	BT-270	86	4.7	4.4	0.3	17	*	< LOD	–	8
750	BT-192	164	5.1	3.9	0.6	8	7.1	< LOD	–	6
Real (positive samples), containing *Cannabis* pollen										
780	BT-174	182	4.9	4.5	0.1	53	8.0	25.9	99.2	5
880	BT-150	206	4.6	3.7	0.2	22	10.1	42.6	165.9	
950	BT-145	211	4.9	4.8	0.1	56	*	46.1	171.0	4
1000	BT-120	236	4.8	2.8	0.2	16	6.0	49.4	196.7	
1500	BT-102	254	4.8	3.9	0.4	11	7.6	88.8	348.5	
2200	BT-96	260	*	2.5	0.3	10	*	123.6	486.5	
2560	BT-91	265	5.0	2.3	0.2	13	5.8	70.1	271.7	3
2720	BT-85	271	*	1.3	0.2	8	*	37.0	145.5	
3020	BT-78	278	5.0	1.0	0.1	12	5.4	< LOQ	–	
Negative sample, without *Cannabis* pollen										
4200	BT-36	320	4.8	2.6	0.1	30	*	*	*	2

*Limited amount of sample

Furthermore, C and N contents were determined to estimate the content of organic matter of the sample material as well as the C/N ratios of organic matter. For investigated sediment samples, C contents correspond approximately to the total organic carbon (TOC) concentration due to pH values between 4.6 and 5.0 and the resulting absence of carbonate carbon. With C contents mostly between 3 and 4%, studied samples were rather rich in organic matter. Measured loss on ignition values at 550 °C (LOI_550_), as a further parameter for estimating the organic content of the sediment samples (see Heiri et al. [19]), confirmed this finding. C/N ratios of 20 and greater indicating organic matter rather from vascular land plants than from phytoplankton (see Meyers and Teranes [24]) and match with findings of Demske et al. [15] (admixtures of plant fragments and woody material were described). C/N values of 8–13 point to organic matter from phytoplankton or a nearly equal mixture of algal and vascular plant contributions. For further geochemical parameters of sediment samples from Badanital lake or discussions concerning past vegetation or climatic changes, see Demske et al. and Kotlia and Joshi [15, 18].

**Fig. 6:**
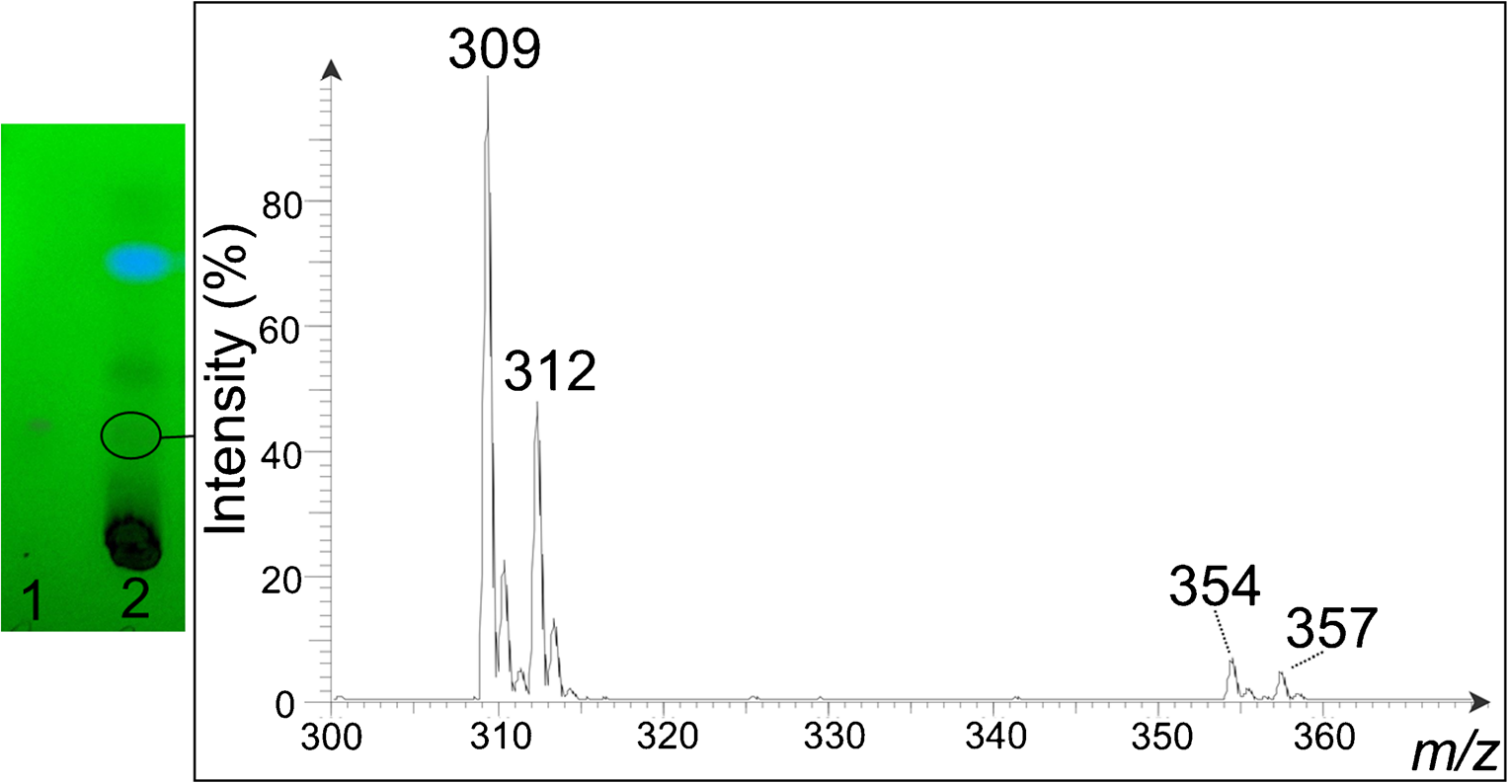
HPTLC chromatogram of (1) CBN standard (190 ng CBN/HPTLC zone) and (2) an extract of real sample BT-102 spiked with CBN-d_3_ (62.5 ng CBN-d_3_/HPTLC zone) as well as a related MS spectrum (HPTLC silica gel 60 plate developed in *n*-heptane/diethyl ether (90:10 v/v); observed with an UV light source at 254 nm; extracting agent: methanol/hexane (10 mL, 9:1 v/v); after an orthogonal SPE sample preparation)

The CBN content of the investigated real samples varied between 99.2 and 486.5 ng CBN/g sediment with a maximum content in PZ 4 at a depth of 260 cm. A characteristic HPTLC chromatogram of an extract and the corresponding MS spectrum is shown in Fig. 6. The data set indicates a correlation with the pollen records of Demske et al. [15]. A high pollen concentration of *Cannabis* type was also found in PZ 3 (293–262 cm; approximately 1620–480 BC) and 4 (262–209 cm; approximately 480 BC–1050 AD), followed by a decreasing contribution of *Cannabis* type in PZ 5 (209–173 cm; approximately 1050–1160 AD). The high percentages of *Cannabis* pollen were interpreted as an indicator of water retting of hemp and the confidential interval of intense retting at Badanital was dated from approximately 480 BC to 1050 AD. This hypothesis is largely corroborated by the record of sedimentary CBN. However, a systematic analysis of sediment core samples from Badanital lake is needed for a detailed discussion of the CBN content with regard to ancient retting activities. This remains the goal of additional investigations to be performed in the future.

## Conclusion

In this paper, we report on the application of HPTLC-ESI-MS to identify and quantify the cannabinoid CBN, an unequivocal molecular biomarker for the *Cannabis* species and consequently a tracer for ancient water retting of *Cannabis* in sediment samples. In the course of method development, planar chromatographic separation, sample extraction, and the subsequent cleanup procedure, using an orthogonal SPE sample preparation, were optimized. To evaluate the potential of this method, parameter such as LOD, LOQ, linearity, recovery rate, method precision, and storage stability were determined. The validated method showed a satisfactory overall analytical performance and determined CBN contents of sediment samples from a small lake in Northern India match very well with pollen records reported in previous studies. In addition, different spray reagents for a post-chromatographic detection of CBN were tested. FBS reagent enables, under the selected conditions, a sensitive and specific detection of CBN in sediment samples.

In conclusion, HPTLC-ESI-MS is a relatively simple, rapid method enabling a high-throughput and low-cost screening of complex and challenging sediment samples as a natural archive for environmental changes and human activities. Considering the still fragmentary knowledge on ancient retting sites and fact that pollen records reflect the presence of *Cannabis*, however, not really the retting of *Cannabis*, this method is a promising approach to track more specifically the retting processes for reconstructing the *Cannabis* retting history.

## Electronic supplementary material

ESM 1 (DOCX 1200 kb)
