# Supplementary material for: Identification and quantification of cannabinol as a biomarker for local hemp retting in an ancient sedimentary record by HPTLC-ESI-MS
Source: Anal Bioanal Chem. 2020 Feb 14;412(11):2633–44. doi: 10.1007/s00216-020-02492-0 (PMC7136313; doi:10.1007/s00216-020-02492-0)
Supplement: Supplementary file 1 — (DOCX 1200 kb) [file 216_2020_2492_MOESM1_ESM.docx]

Analytical and Bioanalytical Chemistry

Electronic Supplementary Material

**Identification and quantification of cannabinol as a biomarker for local hemp retting in an ancient sedimentary record by HPTLC-ESI-MS**

Theresa Schmidt, Annemarie Elisabeth Kramell, Florian Oehler, Ralph Kluge,
Dieter Demske, Pavel E Tarasov, René Csuk

**Table of content**

Figure S1 Calibration curve obtained from CBN in the range 25 – 155 ng CBN/HPTLC zone.

Figure S2 MS spectrum of a mixture of CBN and CBN-d_3_ spotted onto a HPTLC silica gel 60 plate recorded (a) immediately after chromatographic separation and (b) after 3 h.

Figure S3 MS spectrum of CBD and CBD-d_3_ spotted onto a HPTLC silica gel 60 plate using *n*-heptane/diethyl ether (90:10 v/v)] as developing solvent.

Figure S4 TLC silica gel 60 plates developed using (a) n‑heptane/diethyl ether/formic acid (75:25:0.3 v/v/v) and (b) n-hexane/acetone/triethylamine (40:20:2 v/v/v) as developing solvent; observed with an UV light source at 254 nm.

Figure S5 Variation of CBN concentration in working solutions of CBN stored at ‑12 °C in the dark, at room temperature in the dark and at room temperature exposed to sunlight, over a period of 31 days. Initial concentration of CBN (a) 2.2 µg/mL; (b) 5.0 µg/mL.

Table S1 CBN concentration of calibration solutions and related average peak area ratios of CBN and CBN-d_3_ received from analyses on calibration solutions in triplicate after chromatographic separation.

Table S2 Data set for experiments concerning the precision.

Table S3 Data set for experiments concerning the trueness.

Table S4 Recovery of the method determined using negative samples spiked with CBN and characteristics of these samples.

Table S5 Data set for experiments concerning the storage stability of CBN standard solutions.

**
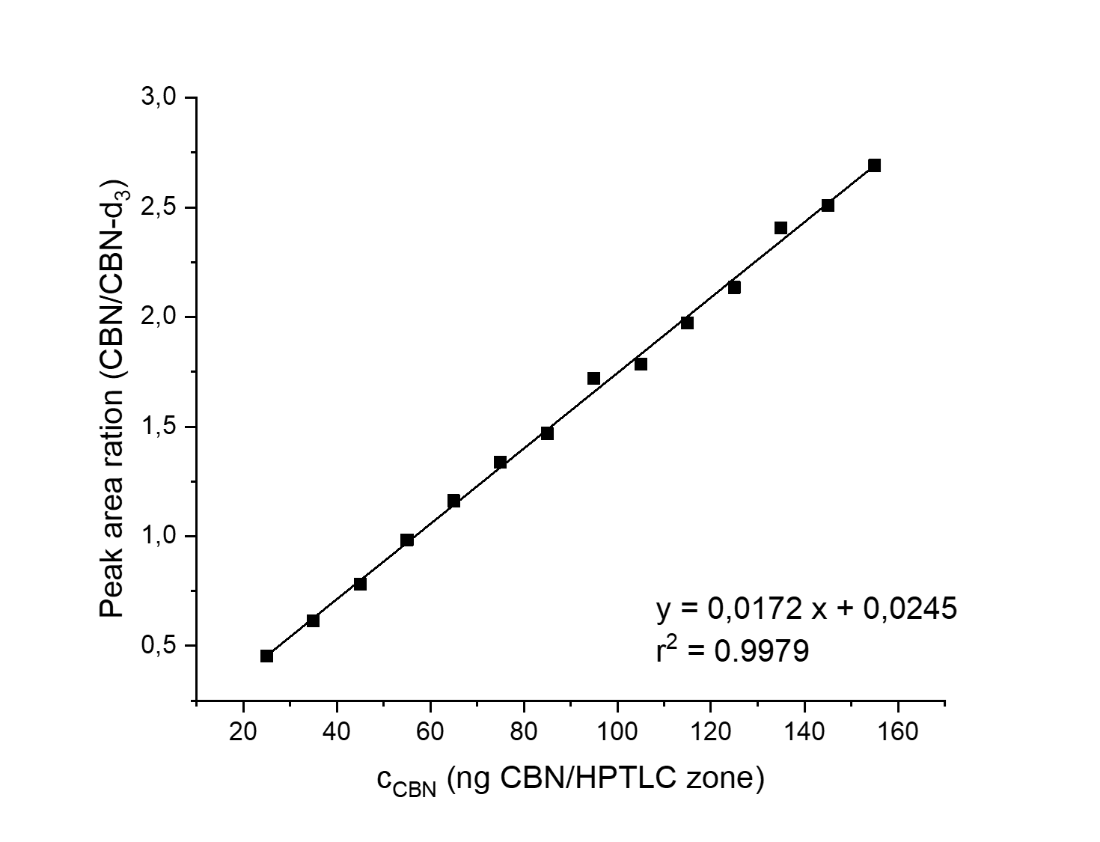
**

**Fig. S1** Calibration curve obtained from CBN in the range 25 – 155 ng CBN/HPTLC zone with 62,5 ng CBN‑d_3_/HPTLC zone after chromatographic separation

**Tab. S1** CBN concentration of calibration solutions and related average peak area ratios of CBN (sum of mass peaks at m/z 309 and 354) and CBN-d_3_ (sum of mass peaks at m/z 312 and 357) received from analyses on calibration solutions in triplicate (25 - 155 ng CBN/HPTLC zone; 62,5 ng CBN-d_3_/HPTLC zone) after chromatographic separation

| **c_CBN_  (ng CBN/HPTLC zone)** | **Average peak area ratio**  **(∑ *m/z*309+354)/(∑ *m/z* 312+357)** |
| --- | --- |
| 25 | 0.4534 |
| 35 | 0.6139 |
| 45 | 0.7816 |
| 55 | 0.9831 |
| 65 | 1.1609 |
| 75 | 1.3379 |
| 85 | 1.4685 |
| 95 | 1.7203 |
| 105 | 1.7835 |
| 115 | 1.9735 |
| 125 | 2.1344 |
| 135 | 2.4072 |
| 145 | 2.5084 |
| 155 | 2.6911 |

**Tab. S2** Data set for experiments concerning the precision

| **Repeatability** (n = 24; measured value classified as outliner by Dixon's test was rejected) | |
| --- | --- |
| **Key figure** | **Result** |
| Mean (ng CBN/HPTLC zone) | 88.76 |
| SD (standard deviation in ng CBN/HPTLC zone) | 3.82 |
| RSD (relative standard deviation in %) | 4.30 |
| Results of Neumann trend test | |
| Test value | 30.80 |
| Characteristic value (99 %) | 1.11 |
| No trend was observed with a statistical safety of 99 % | |
| **Method precision** **using sediment sample BT-78** (n = 6; no measured value was classified as outliner by Dixon's test) | |
| **Key figure** | **Result** |
| Mean (ng CBN/HPTLC zone) | 131.90 |
| SD (standard deviation in ng CBN/HPTLC zone) | 5.36 |
| RSD (relative standard deviation in %) | 4.06 |
| Results of Neumann trend test | |
| Test value | 2.69 |
| Characteristic value (99 %) | 0.58 |
| No trend was observed with a statistical safety of 99 % | |

**Tab. S3** Data set for experiments concerning the trueness

| **Bias calculation with sediment sample BT-102**  (spiked with CBN 100 µL, 5.4 µg/ml, i.e. 135 ng CBN/HPTLC zone; average CBN content of the untreated sediment sample: 88.8 ng CBN/HPTLC zone) | |
| --- | --- |
| **Key figure** | **Result** |
| Mean (ng CBN/HPTLC zone) | 237.89 |
| True concentration (ng CBN/HPTLC zone) | 223.8 |
| Bias (ng CBN/HPTLC zone) | 14.09 |
| Results of Student’s t-test | |
| SD (standard deviation in ng CBN/HPTLC zone) | 15.85 |
| Test value | 1.54 |
| Critical value [t(2;0.99)] | 9.93 |
| no statistical difference between the mean and the “true” values | |
| **Bias calculation with sediment sample BT-145**  (spiked with CBN 100 µL, 1.8 µg/ml, i.e. 45 ng CBN/HPTLC zone; average CBN content of the untreated sediment sample: 46.1 ng CBN/HPTLC zone) | |
| **Key figure** | **Result** |
| Mean (ng CBN/HPTLC zone) | 104.83 |
| True concentration (ng CBN/HPTLC zone) | 91.1 |
| Bias (ng CBN/HPTLC zone) | 13.73 |
| Results of Student’s t-test | |
| SD (standard deviation in ng CBN/HPTLC zone) | 2.90 |
| Test value | 8.20 |
| Critical value [t(2;0.99)] | 9.93 |
| no statistical difference between the mean and the “true” values | |

**Tab. S4** Recovery of the method determined using negative samples spiked with CBN and characteristics of these samples (62,5 ng CBN-d_3_/HPTLC zone). Two aliquotes of each sediment sample were used for calculation

| **Estimated ages (ca cal yrs BP)** | **Sample ID** | **Depth in sedimentary core (cm)** | **Calculated CBN content**  **(ng CBN/HPTLC zone** | **Calculated CBN content**  **(ng CBN/g sediment)** | **Target content of CBN (ng CBN/g sediment)** | **Recovery (%)** |
| --- | --- | --- | --- | --- | --- | --- |
| 380 | BT-270.1 | 86 | 64.6 | 249.3 | 328.2 | 76 |
|  | BT-270.2 |  | 66.1 | 256.4 | 329.8 | 78 |
| 730 | BT-192.1 | 164 | 42.8 | 170.1 | 248.8 | 68 |
|  | BT-192.2 |  | 44.8 | 174.6 | 239.9 | 72 |
| 4250 | BT-36.1 | 320 | 22.1* | 87.9 | 139.2 | 63 |
|  | BT-36.2 |  | 28.6 | 113.7 | 139.0 | 82 |

* below lowest concentration used for calibration; however above LOQ

**Tab. S5** Data set for experiments concerning the storage stability of CBN standard solutions (n = 11)

| **Key figure** | **Result** |
| --- | --- |
| **2.2 µg/mL, RT, light** | |
| Mean (% initial CBN concentration) | 98.26 |
| SD (standard deviation in % initial CBN concentration) | 8.31 |
| Results of Neumann trend test | |
| Test value | 1.97 |
| Characteristic value (99 %) | 0.7915 |
| No trend was observed with a statistical safety of 99 % | |
| **2.2 µg/mL, RT, dark** | |
| Mean (% initial CBN concentration) | 85.97 |
| SD (standard deviation in % initial CBN concentration) | 8.68 |
| Results of Neumann trend test | |
| Test value | 1.84 |
| Characteristic value (99 %) | 0.7915 |
| No trend was observed with a statistical safety of 99 % | |
| **2.2 µg/mL, -12 °C, dark** | |
| Mean (% initial CBN concentration) | 91.04 |
| SD (standard deviation in % initial CBN concentration) | 7.94 |
| Results of Neumann trend test | |
| Test value | 1.45 |
| Characteristic value (99 %) | 0.7915 |
| No trend was observed with a statistical safety of 99 % | |
| **5.0 µg/mL, RT, light** | |
| Mean (% initial CBN concentration) | 103.48 |
| SD (standard deviation in % initial CBN concentration) | 6.17 |
| Results of Neumann trend test | |
| Test value | 1.62 |
| Characteristic value (99 %) | 0.7915 |
| No trend was observed with a statistical safety of 99 % | |
| **5.0 µg/mL, RT, dark** | |
| Mean (% initial CBN concentration) | 104.39 |
| SD (standard deviation in % initial CBN concentration) | 9.12 |
| Results of Neumann trend test | |
| Test value | 1.73 |
| Characteristic value (99 %) | 0.7915 |
| No trend was observed with a statistical safety of 99 % | |
| **5.0 µg/mL, -12 °C, dark** | |
| Mean (% initial CBN concentration) | 97.76 |
| SD (standard deviation in % initial CBN concentration) | 9.14 |
| Results of Neumann trend test | |
| Test value | 2.07 |
| Characteristic value (99 %) | 0.7915 |
| No trend was observed with a statistical safety of 99 % | |


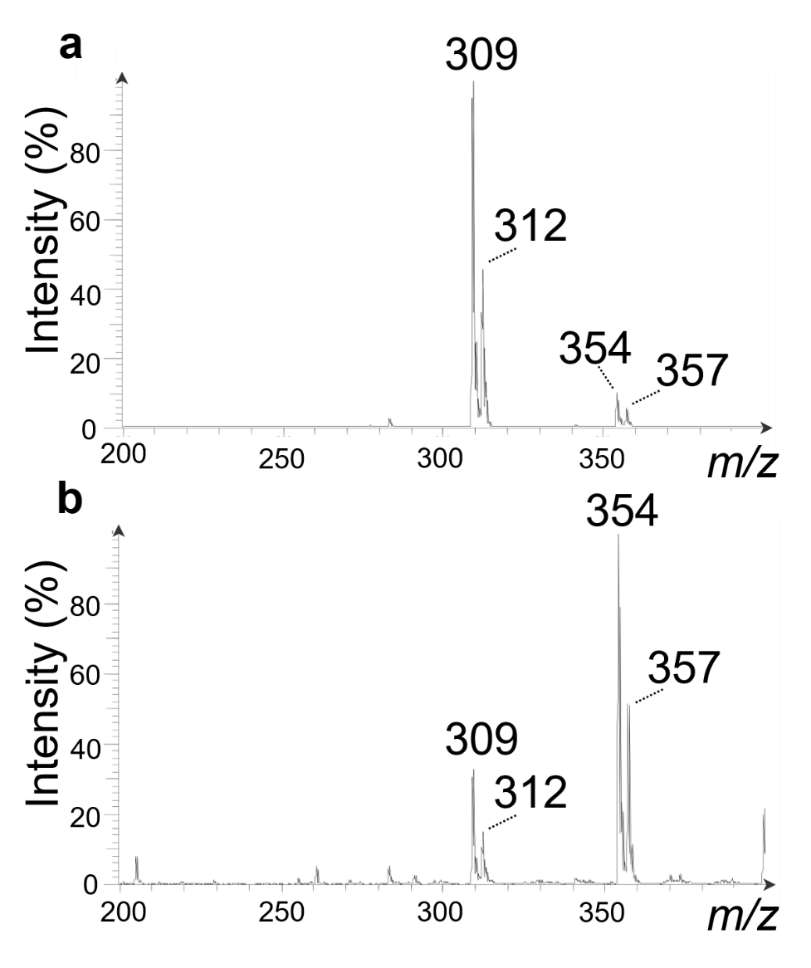


**Fig. S2** MS spectrum of a mixture of CBN and CBN-d_3_ spotted onto a HPTLC silica gel 60 plate recorded (a) immediately after chromatographic separation and (b) after 3 h [developing solvent: *n*-heptane/diethyl ether (90:10 v/v)]


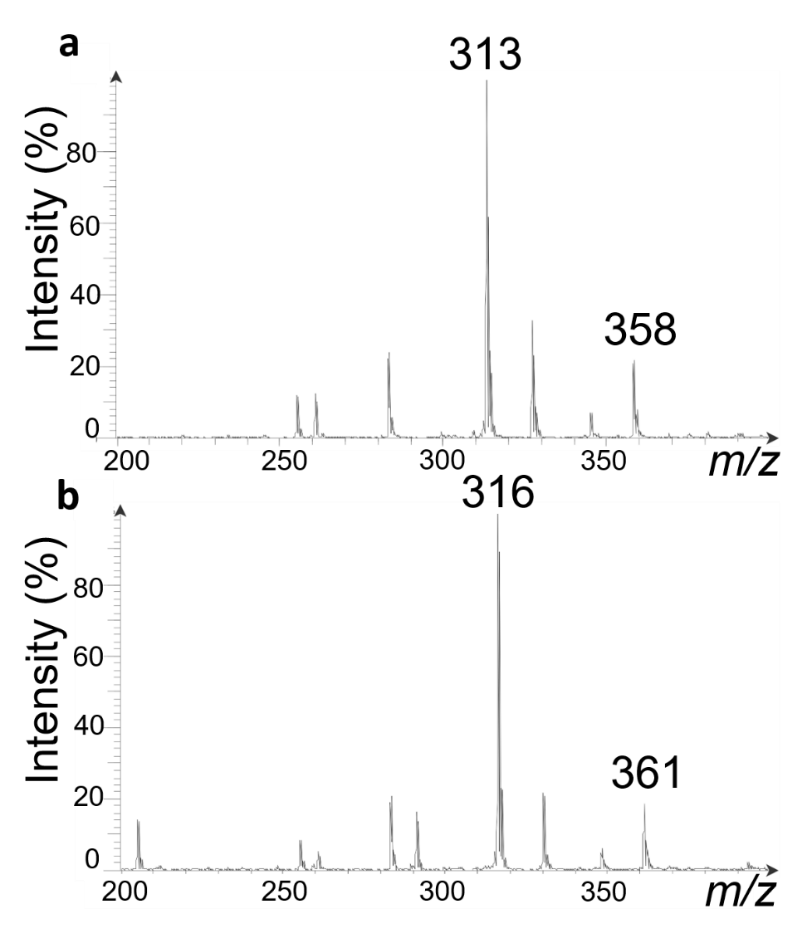


**Fig. S3** MS spectrum of (a) CBD and (b) CBD-d_3_ spotted onto a HPTLC silica gel 60 plate using *n*-heptane/diethyl ether (90:10 v/v)] as developing solvent

**
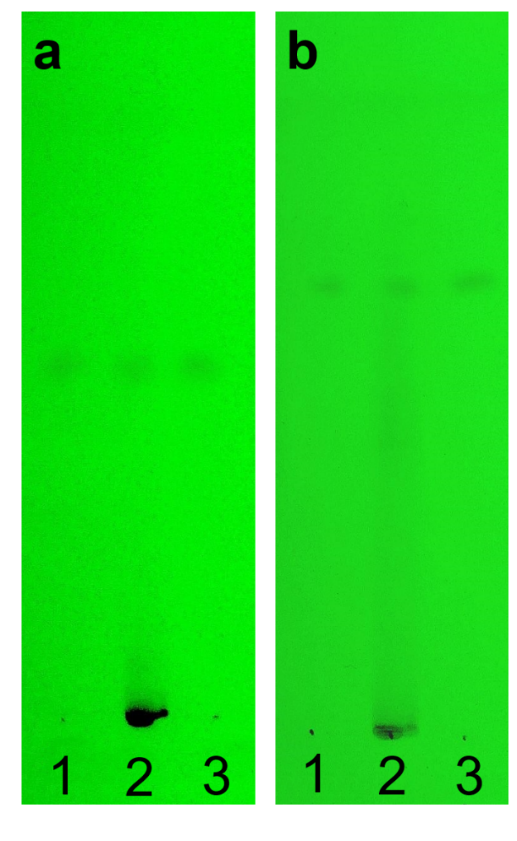
**

**Fig. S4** TLC silica gel 60 plates developed using (a) *n*‑heptane/diethyl ether/formic acid (75:25:0.3 v/v/v) and (b) *n*-hexane/acetone/triethylamine (40:20:2 v/v/v) as developing solvent; observed with an UV light source at 254 nm. Tracks: 1 = CBN standard (62,5ng CBN/TLC zone; 125 ng CBN-d_3_/TLC zone); 2 = diluted extract [extracting agent: dichloromethane/methanol (1:1 v/v); without a SPE sample preparation] of negative sample BT-270 spiked with CBN (62.5 ng CBN/TLC zone; 125 ng CBN-d_3_/HPTLC zone); 3 = CBN standard (125 ng CBN/TLC zone; 125 ng CBN-d_3_/TLC zone)


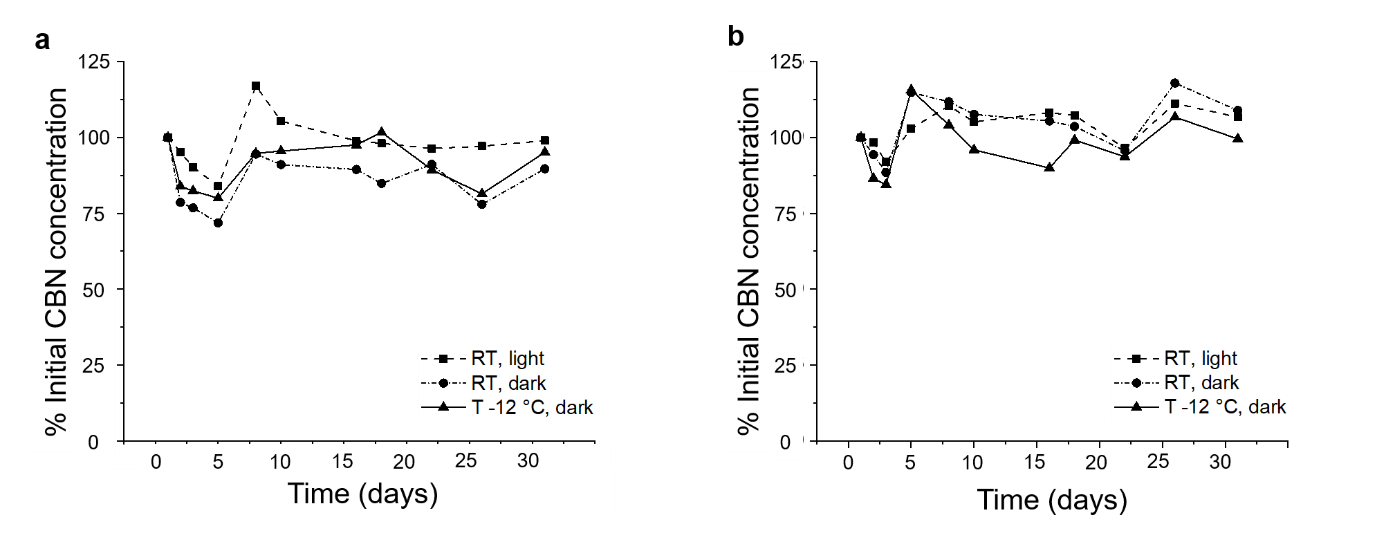


**Fig. S5** Variation of CBN concentration in working solutions of CBN stored at ‑12 °C in the dark, at room temperature in the dark and at room temperature exposed to sunlight, over a period of 31 days. Initial concentration of CBN (a) 2.2 µg/mL; (b) 5.0 µg/mL
